# Prevalence of Self-Reported Hypertension and Antihypertensive Medication Use Among Adults — United States, 2017–2021

**DOI:** 10.15585/mmwr.mm7309a1

**Published:** 2024-03-07

**Authors:** Ahlia Sekkarie, Jing Fang, Donald Hayes, Fleetwood Loustalot

**Affiliations:** 1 Division for Heart Disease and Stroke Prevention, National Center for Chronic Disease Prevention and Health Promotion, CDC.

**Summary**

**What is already known about this topic?**

High blood pressure (hypertension) is a major risk factor for heart disease and stroke. It increases with age and varies by different populations and states. In 2017, updated guidelines recommended lowering the blood pressure threshold for diagnosis of hypertension in adults.

**What is added by this report?**

From 2017 to 2021, approximately one third of U.S. adults reported diagnosed hypertension; prevalence varied by sociodemographic characteristics and state of residence. Among persons reporting hypertension, the prevalence of antihypertensive medication use increased by approximately 3 percentage points.

**What are the implications for public health practice?**

Knowledge of hypertension diagnosis and treatment prevalence and trends can help guide the development of policies and implementation of evidence-based interventions to reduce disparities in this important risk factor for cardiovascular disease.

## Abstract

Hypertension, or high blood pressure, is a major risk factor for heart disease and stroke. It increases with age and is highest among non-Hispanic Black or African American persons, men, persons aged ≥65 years, those of lower socioeconomic status, and those who live in the southern United States. Hypertension affects approximately one half of U.S. adults, and approximately one quarter of those persons have their blood pressure under control. Reducing population-level hypertension prevalence and improving control is a national priority. In 2017, updated guidelines for high blood pressure in adults recommended lowering the blood pressure threshold for diagnosis of hypertension. Analysis of data from the Behavioral Risk Factor Surveillance System found that age-standardized, self-reported diagnosed hypertension was approximately 30% during 2017–2021, with persistent differences by age, sex, race and ethnicity, level of education, and state of residence. During this period, the age-standardized prevalence of antihypertensive medication use among persons with hypertension increased by 3.1 percentage points, from 59.8% to 62.9% (p<0.001). Increases in antihypertensive medication use were observed in most sociodemographic groups and in many states. Assessing current trends in hypertension diagnosis and treatment can help guide the development of policies and implementation of interventions to reduce this important risk factor for cardiovascular disease and can aid in addressing health disparities.

## Introduction

Hypertension, or high blood pressure, is a major risk factor for heart disease and stroke (*1*). Hypertension affects approximately one in two U.S. adults aged ≥18 years, approximately one quarter of whom have their blood pressure under control (*1*). Prevalence of hypertension is highest among non-Hispanic Black or African American (Black) persons, men, persons aged ≥65 years, those of lower socioeconomic status, and those who live in the southern United States (*2*). Improving population-level hypertension prevalence and control is a national priority.* In 2017, updated guidelines for high blood pressure in adults recommended lowering the blood pressure threshold for diagnosis of hypertension (*3*). This change would be expected to lead to increased diagnosed hypertension prevalence. CDC analyzed data from the Behavioral Risk Factor Surveillance System (BRFSS) to examine characteristics and trends in prevalence of self-reported diagnosed hypertension and antihypertensive medication use.

## Methods

### Data Source and Primary Measures

CDC analyzed data from BRFSS, a state-based telephone survey of noninstitutionalized U.S. adults aged ≥18 years.^†^ The median response rates for the 50 states and the District of Columbia in 2017, 2019, and 2021 were 45.8% (range = 30.6%–64.1%), 49.4% (37.3%–73.1%), and 43.8% (23.5%–60.5%), respectively.^§^ Self-reported diagnosed hypertension (hypertension) was defined as an affirmative response to the question, “Have you ever been told by a doctor, nurse, or other health professional that you have high blood pressure?” Respondents who reported that they were told they had blood pressure levels that were borderline high, elevated, prehypertensive, or had high blood pressure only during pregnancy were not classified as having hypertension. To determine whether persons with hypertension were being treated, respondents who answered the first question affirmatively were then asked, “Are you currently taking medicine for your high blood pressure?” Hypertension and treatment were assessed by age group (18–44, 45–64, and ≥65 years), sex (female and male), race and ethnicity (non-Hispanic White [White]; Black; Hispanic or Latino; non-Hispanic Asian [Asian]; non-Hispanic Native Hawaiian or other Pacific Islander [NH/OPI]; non-Hispanic American Indian or Alaska Native [AI/AN]; and non-Hispanic other [other] persons), highest level of education attained (less than high school graduate, high school diploma or general educational development certificate, some college, or college graduate or higher), and state of residence.

### Data Analysis

Prevalence estimates were age-standardized to the 2000 U.S. Census Bureau population using three age groups (18–44, 45–64, and ≥65 years) for all characteristics except age-specific estimates. Prevalence differences (i.e., percentage point differences) between 2017 and 2021 were assessed using t-tests adjusted for sex, age, and race and ethnicity in a logistic regression model. P*-*values <0.05 were considered statistically significant. All analyses were conducted using SAS-callable SUDAAN (version 11.0.4; RTI International) to account for the complex sampling design and weighting. This activity was reviewed by CDC, deemed not research, and was conducted consistent with applicable federal law and CDC policy.^¶^

## Results

During 2017, 2019, and 2021, a total of 444,023, 409,810, and 431,639 participants, respectively, were interviewed. After investigators excluded participants who were pregnant (0.5%–0.6%), missing data for hypertension variables (0.4%–0.5%), and other covariates (3.2%–3.9%), the final analytic samples for 2017, 2019, and 2021 were 425,417 (96% of original sample), 392,100 (96%), and 410,318 (95%), respectively.

### Hypertension Prevalence

From 2017 to 2021, the overall age-standardized prevalence of hypertension did not change, remaining at almost exactly 30% (Table 1). In 2021, hypertension prevalence was higher among men (33.2%) than among women (27.0%), among adults aged ≥65 years (60.6%) than among those aged 18–44 years (14.5%) and 45–64 years (40.3%), among Black adults (40.2%) than among Asian adults (22.7%), and among persons with less than a high school education (33.8%) than among those with some college (31.2%) or a college degree or higher education (25.5%).

**TABLE 1:** Age-standardized prevalence* of hypertension among adults aged ≥18 years, by sociodemographic characteristics and state and the District of Columbia — Behavioral Risk Factor Surveillance System, United States, 2017–2021

Characteristic	Prevalence (95% CI)			2017 vs. 2021	
	2017	2019	2021	Percentage point difference	p-value^†^
**Total**	**30.1 (29.8–30.3)**	**30.0 (29.7–30.2)**	**30.1 (29.8–30.4)**	**0**	**0.890**
**Sex**					
Men	32.9 (32.5–33.4)	33.0 (32.7–33.4)	33.2 (32.8–33.6)	0.3	0.272
Women	27.2 (26.8–27.5)	26.9 (26.6–27.2)	27.0 (26.6–27.4)	–0.2	0.348
**Age group, yrs**					
18–44	14.3 (14.0–14.7)	14.3 (13.9–14.6)	14.5 (14.1–14.9)	0.2	0.333
45–64	40.6 (40.1–41.1)	40.6 (40.1–41.2)	40.3 (39.7–40.8)	–0.3	0.408
≥65	60.5 (59.9–61.1)	60.1 (59.6–60.6)	60.6 (60.0–61.2)	0.1	0.902
**Race and ethnicity^§^**					
AI/AN	37.3 (35.1–39.5)	34.7 (32.5–36.8)	36.5 (34.5–38.5)	–0.8	0.673
Asian	23.7 (21.8–25.7)	23.7 (22.1–25.4)	22.7 (20.8–24.7)	–1.0	0.570
Black or African American	40.0 (39.2–40.9)	39.7 (38.9–40.5)	40.2 (39.3–41.1)	0.2	0.831
NH/OPI	33.3 (29.6–37.3)	30.3 (26.0–34.9)	31.1 (27.2–35.4)	–2.2	0.673
White	29.1 (28.8–29.4)	29.4 (29.1–29.7)	29.3 (29.0–29.6)	0.2	0.351
Hispanic or Latino	28.4 (27.4–29.4)	27.3 (26.4–28.3)	27.5 (26.5–28.6)	–0.9	0.343
Other	30.0 (27.1–33.0)	29.0 (26.8–31.2)	30.1 (27.7–32.7)	0.1	0.954
**Highest level of education attained**					
Less than high school	36.1 (35.1–37.1)	34.9 (34.0–35.9)	33.8 (32.7–34.9)	–2.3	0.006
High school graduate or GED	32.5 (32.0–33.1)	32.5 (32.0–33.0)	32.6 (32.0–33.2)	0.1	0.745
Some college	30.2 (29.7–30.7)	30.3 (29.8–30.8)	31.2 (30.6–31.7)	1.0	0.013
College graduate or higher	24.7 (24.3–25.1)	25.2 (24.9–25.6)	25.5 (25.1–25.9)	0.8	0.004
**Residence**					
Alabama	38.7 (37.2–40.3)	38.9 (37.5–40.3)	38.9 (37.1–40.7)	0.2	0.724
Alaska	32.1 (29.6–34.6)	32.6 (30.1–35.1)	29.4 (27.8–31.2)	–2.6	0.111
Arizona	28.0 (27.2–28.9)	29.7 (28.2–31.3)	28.0 (26.9–29.2)	–0	0.779
Arkansas	38.4 (36.0–40.8)	37.8 (36.0–39.6)	37.4 (35.5–39.2)	–1.0	0.718
California	27.0 (25.9–28.1)	26.6 (25.6–27.6)	26.3 (24.9–27.6)	–0.7	0.335
Colorado	24.3 (23.4–25.2)	24.2 (23.2–25.1)	24.6 (23.7–25.6)	0.3	0.833
Connecticut	27.3 (26.2–28.4)	27.5 (26.3–28.7)	27.8 (26.5–29.1)	0.5	0.704
Delaware	31.4 (29.5–33.4)	32.8 (30.8–34.9)	31.7 (29.7–33.7)	0.3	0.837
District of Columbia	28.3 (26.8–29.8)	29.2 (27.4–31.1)	29.6 (27.8–31.4)	1.3	0.319
Florida^¶^	29.8 (28.6–31.2)	28.5 (27.2–29.9)	—	—	—
Georgia	31.6 (30.2–33.1)	32.7 (31.2–34.3)	34.6 (33.2–36.0)	2.9	0.003
Hawaii	28.3 (27.1–29.7)	27.8 (26.6–29.2)	26.4 (25.1–27.7)	–1.9	0.016
Idaho	27.7 (26.2–29.3)	28.5 (26.8–30.3)	28.2 (27.0–29.4)	0.5	0.802
Illinois	29.9 (28.6–31.4)	29.5 (28.2–30.8)	26.8 (25.0–28.7)	–3.2	0.006
Indiana	32.8 (31.8–33.8)	32.4 (31.2–33.5)	31.8 (30.8–32.9)	–1.0	0.152
Iowa	28.3 (27.2–29.4)	28.9 (27.9–29.9)	28.5 (27.4–29.6)	0.2	0.720
Kansas	30.6 (29.9–31.3)	31.3 (30.3–32.3)	31.6 (30.8–32.4)	1.0	0.080
Kentucky	36.3 (34.8–37.8)	37.6 (35.9–39.2)	36.9 (35.3–38.7)	0.6	0.888
Louisiana	37.1 (35.5–38.7)	37.3 (35.7–38.9)	37.3 (35.6–39.0)	0.2	0.834
Maine	30.0 (28.6–31.5)	30.9 (29.5–32.4)	28.2 (27.0–29.4)	–1.9	0.054
Maryland	30.2 (29.1–31.3)	31.6 (30.6–32.6)	31.7 (30.6–32.7)	1.5	0.100
Massachusetts	25.9 (24.4–27.4)	25.3 (24.2–26.5)	26.2 (25.0–27.5)	0.4	0.783
Michigan	31.5 (30.4–32.6)	31.4 (30.3–32.6)	31.5 (30.4–32.7)	0	0.968
Minnesota	24.4 (23.7–25.2)	26.2 (25.4–26.9)	26.8 (26.0–27.6)	2.4	<0.001
Mississippi	38.2 (36.4–40.1)	40.9 (39.2–42.6)	40.6 (38.8–42.5)	2.4	0.036
Missouri	29.0 (27.8–30.4)	27.8 (26.5–29.2)	32.1 (30.9–33.3)	3.1	0.001
Montana	25.9 (24.5–27.4)	25.7 (24.5–26.9)	27.0 (25.7–28.3)	1.1	0.326
Nebraska	28.5 (27.5–29.5)	28.7 (27.8–29.6)	29.6 (28.6–30.5)	1.1	0.188
Nevada	30.5 (28.5–32.6)	29.9 (27.7–32.2)	29.7 (27.4–32.1)	–0.8	0.480
New Hampshire	26.0 (24.5–27.5)	27.8 (26.2–29.5)	26.1 (24.7–27.5)	0.1	0.710
New Jersey^¶^	30.4 (29.0–31.8)	—	27.5 (26.3–28.8)	–2.9	0.003
New Mexico	28.5 (27.0–30.0)	28.8 (27.3–30.4)	29.8 (28.4–31.4)	1.4	0.238
New York	27.1 (26.1–28.2)	27.0 (26.0–28.0)	27.6 (26.9–28.3)	0.4	0.300
North Carolina	32.0 (30.5–33.6)	32.4 (30.9–34.0)	31.3 (29.9–32.8)	–0.7	0.515
North Dakota	28.3 (27.1–29.5)	28.2 (26.7–29.7)	29.3 (27.9–30.7)	1.0	0.367
Ohio	31.7 (30.5–32.9)	31.2 (30.0–32.4)	32.0 (31.0–33.1)	0.4	0.527
Oklahoma	35.4 (34.0–36.8)	35.5 (34.1–36.8)	37.1 (35.5–38.7)	1.7	0.178
Oregon	27.5 (26.2–28.8)	27.6 (26.3–28.9)	27.5 (26.2–28.9)	0	0.816
Pennsylvania	28.8 (27.5–30.2)	29.4 (28.1–30.7)	29.6 (28.3–31.0)	0.8	0.326
Rhode Island	30.0 (28.4–31.6)	30.3 (28.7–32.0)	29.5 (28.0–31.1)	–0.5	0.509
South Carolina	34.6 (33.4–35.8)	34.7 (33.3–36.1)	34.0 (32.8–35.3)	–0.6	0.416
South Dakota	28.0 (26.2–29.8)	28.1 (26.1–30.1)	30.5 (28.0–33.1)	2.5	0.093
Tennessee	35.6 (34.0–37.4)	35.9 (34.4–37.4)	34.4 (32.8–36.1)	–1.2	0.346
Texas	32.5 (30.8–34.2)	30.8 (29.5–32.2)	31.8 (30.3–33.4)	–0.6	0.637
Utah	25.6 (24.6–26.6)	26.6 (25.6–27.5)	27.0 (26.0–28.0)	1.5	0.032
Vermont	26.0 (24.7–27.3)	26.1 (24.6–27.6)	25.6 (24.2–27.0)	–0.4	0.434
Virginia	30.4 (29.2–31.6)	31.0 (29.9–32.1)	31.4 (30.3–32.5)	1.0	0.136
Washington	27.8 (26.8–28.8)	28.4 (27.5–29.3)	27.6 (26.6–28.6)	–0.2	0.879
West Virginia	38.9 (37.3–40.5)	38.6 (36.9–40.3)	38.1 (36.7–39.6)	–0.7	0.364
Wisconsin	27.9 (26.3–29.4)	27.7 (26.2–29.3)	27.9 (26.5–29.4)	0.1	0.660
Wyoming	28.4 (26.9–29.9)	27.8 (26.1–29.6)	26.8 (25.0–28.6)	–1.6	0.118

**Abbreviations:** AI/AN = American Indian or Alaska Native; BRFSS = Behavioral Risk Factor Surveillance System; GED = general educational development certificate; NH = non-Hispanic; NH/OPI = Native Hawaiian or other Pacific Islander.

* Directly standardized to the 2000 U.S. Census Bureau standard population.

^†^ Adjusted for sex, age group, and race and ethnicity.

^§^ Persons of Hispanic or Latino (Hispanic) origin might be of any race but are categorized as Hispanic; all racial groups are non-Hispanic. The "other" category includes participants of multiple racial and ethnicity groups.

^¶^ New Jersey in 2019 and Florida in 2021 were unable to collect enough BRFSS data to meet the minimum requirements for inclusion in the BRFSS public-use data set.

Although the overall prevalence of hypertension remained unchanged, among persons with less than high school education, hypertension prevalence declined from 36.1% in 2017 to 33.8% in 2021 (p = 0.006). In contrast, a small but statistically significant increase in hypertension prevalence was observed among persons with some college (from 30.2% to 31.2%; p = 0.013) and among persons with college degrees or higher education (from 24.7% to 25.5%; p = 0.004).

By state, the age-standardized prevalence of hypertension ranged from 24.6% in Colorado to 40.6% in Mississippi in 2021. From 2017 to 2021, increases in the prevalence of hypertension were observed in five states (Georgia, Minnesota, Mississippi, Missouri, and Utah) and decreases were observed in three states (Hawaii, Illinois, and New Jersey). Hypertension prevalence was, in general, higher in southeastern and Appalachian states and lower in western states (Figure).

**FIGURE:**
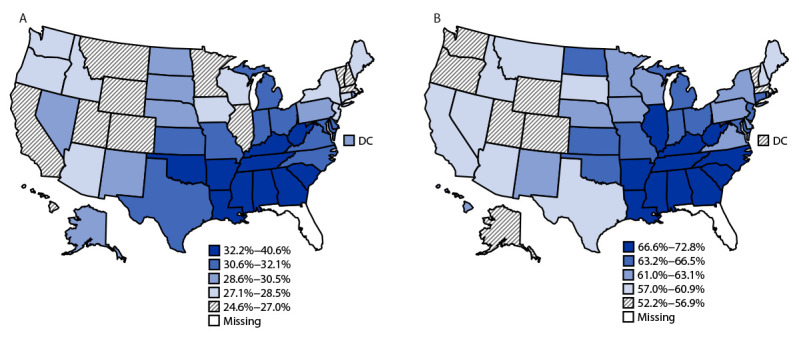
Age-standardized prevalence* of self-reported diagnosed hypertension among adults (A) and use of antihypertensive medication among adults with hypertension (B), by state and the District of Columbia — Behavioral Risk Factor Surveillance System, United States, 2021 **Abbreviation**: DC = District of Columbia. * Data are categorized as quintiles. In 2021, Florida was unable to collect enough Behavioral Risk Factor Surveillance System data to meet the minimum requirements for inclusion in the Behavioral Risk Factor Surveillance System public-use data set.

### Antihypertensive Medication Use

From 2017 to 2021, age-standardized prevalence of antihypertensive medication use among adults with self-reported hypertension increased by 3.1 percentage points, from 59.8% to 62.9% (p<0.001) (Table 2). In 2021, the prevalence of medication use was higher among women (68.5%) than among men (59.4%), among adults aged ≥65 years (92.5%) than among those aged 18–44 years (42.5%), and among Black (71.3%) than among White adults (62%).

**TABLE 2:** Age-standardized prevalence* of antihypertensive medication use among adults aged ≥18 years with hypertension, by sociodemographic characteristics and state and the District of Columbia — Behavioral Risk Factor Surveillance System, United States, 2017–2021

Characteristic	Prevalence (95% CI)			2017 vs. 2021	
	2017	2019	2021	Percentage point difference	p-value^†^
**Total**	**59.8 (59.0–60.5)**	**59.6 (58.9–60.3)**	**62.9 (62.1–63.7)**	**3.1**	**<0.001**
**Sex**					
Men	56.8 (55.9–57.7)	56.7 (55.8–57.6)	59.4 (58.5–60.3)	2.6	<0.001
Women	64.4 (63.1–65.6)	64.3 (63.1–65.4)	68.5 (67.1–69.8)	4.1	<0.001
**Age group, yrs**					
18–44	38.0 (36.7–39.4)	37.7 (36.4–39.0)	42.5 (41.1–44.0)	4.5	<0.001
45–64	80.0 (79.3–80.6)	80.0 (79.3–80.7)	82.2 (81.5–82.9)	2.2	<0.001
≥65	91.9 (91.5–92.4)	92.1 (91.7–92.4)	92.5 (92.1–93.0)	0.6	0.061
**Race and ethnicity^§^**					
AI/AN	58.7 (53.9–63.3)	63.4 (59.0–67.7)	64.0 (59.4–68.3)	5.3	0.073
Asian	58.8 (53.6–63.7)	61.1 (56.1–65.8)	65.7 (60.2–70.9)	6.9	0.167
Black or African American	67.9 (66.0–69.7)	67.4 (65.5–69.3)	71.3 (69.4–73.1)	3.3	0.002
NH/OPI	53.2 (46.2–60.0)	63.7 (54.6–71.9)	62.0 (53.4–69.8)	8.8	0.191
White	58.9 (58.1–59.8)	57.9 (57.1–58.7)	62.0 (61.2–62.9)	3.1	<0.001
Hispanic or Latino	54.3 (52.1–56.5)	56.3 (54.2–58.5)	56.0 (53.7–58.4)	1.8	0.501
Other	56.3 (48.5–63.7)	58.1 (52.6–63.4)	57.1 (51.3–62.6)	0.8	0.706
**Highest level of education attained**					
Less than high school	59.4 (57.1–61.7)	57.6 (55.5–59.7)	60.6 (57.9–63.3)	1.2	0.868
High school graduate or GED	59.7 (58.4–61.0)	59.4 (58.1–60.6)	62.4 (60.9–63.9)	2.7	<0.001
Some college	59.7 (58.4–61.0)	60.8 (59.4–62.1)	63.9 (62.4–65.3)	4.2	<0.001
College graduate or higher	60.1 (58.7–61.5)	59.5 (58.3–60.6)	63.4 (62.2–64.6)	3.3	<0.001
**Residence**					
Alabama	70.1 (66.6–73.4)	70.7 (67.4–73.8)	70.8 (66.5–74.8)	0.7	0.216
Alaska	52.8 (46.4–59.2)	45.5 (41.0–50.0)	54.3 (49.8–58.7)	1.5	0.395
Arizona	56.6 (54.2–59.0)	55.2 (51.0–59.2)	57.1 (54.0–60.2)	0.5	0.578
Arkansas	69.5 (64.0–74.4)	65.1 (60.8–69.1)	66.9 (62.3–71.1)	–2.6	0.609
California	52.9 (49.8–56.0)	53.5 (50.7–56.3)	57.3 (53.2–61.4)	4.4	0.142
Colorado	52.6 (49.5–55.8)	50.5 (47.4–53.6)	54.3 (51.5–57.0)	1.6	0.522
Connecticut	56.9 (53.5–60.2)	57.0 (53.4–60.6)	63.2 (59.3–67.0)	6.3	0.011
Delaware	59.2 (53.5–64.6)	60.1 (54.6–65.4)	62.1 (57.0–67.1)	3.0	0.443
District of Columbia	62.2 (57.7–66.5)	58.4 (52.7–63.9)	54.1 (49.5–58.6)	–8.1	0.166
Florida^¶^	58.5 (55.0–62.0)	59.2 (55.0–63.2)	—	—	—
Georgia	63.6 (59.6–67.4)	62.5 (58.4–66.5)	69.5 (65.7–73.1)	5.9	0.126
Hawaii	57.9 (54.2–61.5)	54.7 (51.0–58.4)	62.6 (58.2–66.8)	4.7	0.052
Idaho	48.7 (44.8–52.6)	54.8 (50.4–59.0)	57.0 (53.5–60.4)	8.3	0.007
Illinois	60.1 (55.6–64.4)	54.3 (50.8–57.7)	67.1 (61.5–72.3)	7.0	0.001
Indiana	60.5 (57.8–63.1)	64.8 (61.5–68.0)	66.5 (63.6–69.3)	6.0	<0.001
Iowa	60.7 (57.4–64.0)	61.8 (59.0–64.6)	62.4 (59.3–65.5)	1.7	0.088
Kansas	59.5 (57.5–61.4)	59.3 (56.8–61.7)	65.8 (63.6–67.9)	6.3	<0.001
Kentucky	67.6 (64.1–70.9)	69.3 (65.7–72.8)	69.3 (65.7–72.6)	1.7	0.106
Louisiana	69.1 (65.2–72.7)	64.5 (60.7–68.2)	70.0 (65.9–73.8)	0.9	0.593
Maine	56.4 (52.1–60.7)	53.1 (49.4–56.7)	58.5 (55.1–61.8)	2.1	0.050
Maryland	62.6 (58.9–66.0)	63.1 (60.3–65.9)	63.9 (61.0–66.7)	1.3	0.805
Massachusetts	59.1 (53.5–64.5)	57.5 (53.8–61.1)	55.8 (51.6–60.0)	–3.3	0.279
Michigan	59.5 (56.5–62.3)	58.8 (55.7–61.8)	65.1 (61.9–68.2)	5.7	0.010
Minnesota	58.5 (55.8–61.1)	57.0 (54.7–59.4)	61.4 (58.9–63.8)	2.9	0.028
Mississippi	72.3 (67.8–76.4)	69.7 (66.0–73.2)	72.8 (68.4–76.8)	0.5	0.539
Missouri	63.4 (59.1–67.4)	58.5 (54.6–62.3)	64.3 (61.1–67.3)	0.9	0.550
Montana	51.8 (47.5–56.1)	52.3 (48.3–56.2)	60.5 (56.4–64.4)	8.7	0.021
Nebraska	61.1 (57.9–64.3)	58.8 (56.1–61.4)	63.1 (60.3–65.9)	2.0	0.157
Nevada	55.4 (49.3–61.3)	51.7 (45.6–57.7)	57.4 (51.2–63.3)	2.0	0.092
New Hampshire	62.3 (55.9–68.3)	57.4 (52.5–62.1)	60.8 (55.7–65.7)	–1.5	0.529
New Jersey^¶^	59.0 (55.1–62.8)	—	64.3 (60.3–68.2)	5.3	0.012
New Mexico	56.2 (51.8–60.5)	58.7 (54.3–63.0)	61.2 (57.2–65.0)	5.0	0.328
New York	56.8 (53.8–59.7)	62.0 (58.7–65.2)	62.3 (60.0–64.5)	5.5	0.001
North Carolina	63.3 (58.9–67.5)	58.9 (55.0–62.7)	68.0 (63.9–71.8)	4.7	0.505
North Dakota	63.3 (59.1–67.3)	59.0 (54.5–63.5)	64.3 (59.9–68.4)	0.9	0.438
Ohio	61.2 (58.0–64.3)	57.8 (54.7–60.9)	63.8 (61.0–66.6)	2.6	0.255
Oklahoma	64.6 (61.0–68.0)	63.8 (60.4–67.2)	64.4 (60.7–67.9)	–0.2	0.822
Oregon	53.9 (50.1–57.7)	55.6 (51.8–59.3)	55.3 (51.8–58.7)	1.4	0.566
Pennsylvania	61.1 (57.0–65.0)	60.9 (57.1–64.6)	62.0 (58.4–65.5)	0.9	0.442
Rhode Island	65.5 (60.1–70.6)	60.9 (55.9–65.7)	66.2 (61.5–70.6)	0.6	0.473
South Carolina	69.2 (65.8–72.5)	66.1 (62.5–69.6)	70.2 (66.6–73.5)	1.0	0.108
South Dakota	64.7 (58.6–70.3)	55.5 (50.1–60.8)	59.0 (52.9–64.9)	–5.7	0.976
Tennessee	65.6 (61.2–69.6)	64.4 (60.8–68.0)	70.3 (66.3–74.1)	4.8	0.026
Texas	58.0 (53.8–62.1)	63.1 (59.2–66.8)	60.9 (57.0–64.7)	2.9	0.102
Utah	52.5 (49.7–55.4)	50.8 (48.4–53.3)	52.2 (49.7–54.7)	–0.4	0.519
Vermont	51.8 (47.8–55.7)	54.9 (50.0–59.8)	53.3 (48.9–57.6)	1.5	0.909
Virginia	58.7 (55.4–62.0)	61.7 (58.6–64.7)	63.0 (59.7–66.0)	4.2	0.074
Washington	54.3 (51.6–57.0)	52.1 (49.6–54.6)	53.2 (50.6–55.7)	–1.1	0.925
West Virginia	62.1 (58.7–65.5)	67.0 (63.2–70.7)	69.6 (66.3–72.8)	7.5	0.054
Wisconsin	57.1 (52.5–61.7)	56.9 (52.1–61.7)	61.5 (56.2–66.6)	4.4	0.806
Wyoming	53.5 (49.2–57.7)	49.8 (45.1–54.5)	56.3 (51.1–61.4)	2.8	0.364

**Abbreviations:** AI/AN = American Indian or Alaska Native; BRFSS = Behavioral Risk Factor Surveillance System; GED = general educational development certificate; NH = non-Hispanic; NH/OPI = Native Hawaiian or other Pacific Islander.

* Directly standardized to the 2000 U.S. Census Bureau standard population.

^†^ Adjusted for sex, age group, and race and ethnicity.

^§^ Persons of Hispanic or Latino (Hispanic) origin might be of any race but are categorized as Hispanic; all racial groups are non-Hispanic. The "other" category includes participants of multiple racial and ethnicity groups.

^¶^ New Jersey (2019) and Florida (2021) were unable to collect sufficient BRFSS data to meet the minimum requirements for inclusion in the BRFSS public-use data set.

From 2017 to 2021, increases in antihypertensive medication use among persons with hypertension were reported among both men and women, persons aged 18–44 and 45–64 years, White adults, Black adults, and persons at all education levels except among those with less than a high school education, among whom medication use prevalence did not change.

By state, the prevalence of medication use among persons with reported hypertension ranged from 52.2% in Utah to 72.8% in Mississippi in 2021. Antihypertensive medication use increased in 11 states and did not decrease significantly in any state. In general, similar to the prevalence of hypertension, the prevalence of medication use among persons with hypertension was higher in southeastern and Appalachian states and lower in western states (Figure).

## Discussion

Among U.S. adults, the age-standardized prevalence of self-reported diagnosed hypertension remained stable at approximately 30% from 2017 to 2021. Among persons with self-reported hypertension, reported antihypertensive medication use increased by approximately 3 percentage points from 2017 to 2021. Prevalences of hypertension and antihypertensive medication use among persons with hypertension differed by age, sex, race and ethnicity, education, and state of residence.

The 2017 Guideline for High Blood Pressure in Adults recommended lowering the blood pressure threshold for diagnosis of hypertension from ≥140 mmHg (systolic) to ≥130 mmHg, and from ≥90 mmHg (diastolic) to ≥80 mmHg (*3*). Significant increases in diagnosed hypertension prevalence would be anticipated with lower thresholds for diagnosis (*4*); however, despite this lower threshold, the prevalence of self-reported diagnosed hypertension did not change between 2017 and 2021. Using these lower thresholds for the diagnosis of hypertension (*3*), approximately one half of adults aged ≥18 years had hypertension during 2017–2020 (*1*). However, this analysis found that approximately one third of adults reported a diagnosis of hypertension. Several reasons could account for this finding. First, broad implementation of changes to clinical guidelines takes time, and differing guidelines that use higher thresholds (140/90 mmHg)** might attenuate any changes in diagnosed hypertension prevalence. Second, some clinical performance measures, which serve as tools to advance the translation of guidelines into clinical practice, were not modified to align with the lower thresholds (*5*). For example, the threshold for adequately controlled blood pressure for various insured populations used by one organization remains at the higher threshold of 140/90 mmHg.^††^ In addition, the COVID-19 pandemic might have affected blood pressure levels and diagnosis of hypertension. Early in the COVID-19 pandemic, an increase in measured blood pressure levels was reported in one longitudinal study (*6*). However, self-reported diagnosed hypertension prevalence did not increase among the overall U.S. population, which might have resulted, in part, from fewer visits to health care providers during the pandemic (*7*).

Application of the 2017 Hypertension Guideline was also expected to increase the number of adults who needed to initiate or increase medication to treat hypertension (*8*). Before 2017, reported antihypertensive medication use had been decreasing among persons with hypertension (*9*). Data in this report provide evidence that starting in 2017, antihypertensive medication use increased overall and across most sociodemographic subgroups and many states.

An increase in medication use will likely lead to improved control of hypertension among those treated. BRFSS does not measure hypertension control; however, data from the National Health and Nutrition Examination Survey showed that the prevalence of controlled blood pressure, using the 2017 blood pressure guideline definitions, did not significantly change from 2009–2012 (25.8%) to 2017–2020 (24.3%; p-value trend = 0.417) (*10*).

### Limitations

The findings in this report are subject to at least five limitations. First, results are based on self-reported data, which likely underestimate actual hypertension prevalence. Second, median response rates of <50% across states might limit representatives of the BRFSS sample, resulting in either under- or overestimates of prevalence. However, the application of sampling weights likely reduces the impact of some nonresponse bias. Third, findings do not extend to adults in long-term care facilities, prisons, or those without a telephone, because BRFSS only collects data from noninstitutionalized adults with a landline or mobile telephone. Fourth, New Jersey in 2019 and Florida in 2021 were unable to collect sufficient BRFSS data to meet the minimum requirements for inclusion in the public-use data set; this might further limit the representativeness of the sample. Finally, because of small sample sizes in some demographic categories and jurisdictions, changes in prevalence might not be detectable.

### Implications for Public Health Practice

Using the most recent self-reported state-level hypertension surveillance data, this report found that hypertension remains a significant public health concern with approximately one third of U.S. adults reporting hypertension, and approximately 60% of those persons reporting antihypertensive medication use. These findings can be used to increase awareness of hypertension and promote lifestyle modifications and antihypertensive medication use to optimize blood pressure control and reduce disparities in prevalence and control. Knowledge of trends in diagnosed hypertension and treatment is an essential tool for guiding state-level, individual, clinical, and public health policies and interventions, such as those promoted by the Million Hearts national initiative, to prevent cardiovascular disease.^§§^

## Footnotes

*
https://www.cdc.gov/bloodpressure/docs/SG-CTA-HTN-Control-Report-508.pdf

†
https://www.cdc.gov/brfss

§ https://www.cdc.gov/brfss/annual_data/2021/pdf/2021-DQR-508.pdf

¶ 45 C.F.R. part 46.102(l)(2), 21 C.F.R. part 56; 42 U.S.C. Sect. 241(d); 5 U.S.C. Sect. 552a; 44 U.S.C. Sect. 3501 et seq.

**
https://www.aafp.org/family-physician/patient-care/clinical-recommendations/all-clinical-recommendations/highbloodpressure.html

††
https://www.ncqa.org/hedis/measures/controlling-high-blood-pressure/

§§
https://millionhearts.hhs.gov/about-million-hearts/optimizing-care/bp-control.html
